# Long Noncoding RNAs in Plant Viroids and Viruses: A Review

**DOI:** 10.3390/pathogens9090765

**Published:** 2020-09-18

**Authors:** Nipin Shrestha, Józef J. Bujarski

**Affiliations:** Department of Biological Sciences and Plant Molecular and Bioinformatics Center, Northern Illinois University, DeKalb, IL 60115, USA

**Keywords:** RNA viruses, long noncoding RNAs, defective RNAs, satellite RNAs, subviral RNAs, viroids, RNA interference

## Abstract

Infectious long-noncoding (lnc) RNAs related to plants can be of both viral and non-viral origin. Viroids are infectious plant lncRNAs that are not related to viruses and carry the circular, single-stranded, non-coding RNAs that replicate with host enzymatic activities via a rolling circle mechanism. Viroids interact with host processes in complex ways, emerging as one of the most productive tools for studying the functions of lncRNAs. Defective (D) RNAs, another category of lnc RNAs, are found in a variety of plant RNA viruses, most of which are noncoding. These are derived from and are replicated by the helper virus. D RNA-virus interactions evolve into mutually beneficial combinations, enhancing virus fitness via competitive advantages of moderated symptoms. Yet the satellite RNAs are single-stranded and include either large linear protein-coding ss RNAs, small linear ss RNAs, or small circular ss RNAs (virusoids). The satellite RNAs lack sequence homology to the helper virus, but unlike viroids need a helper virus to replicate and encapsidate. They can attenuate symptoms via RNA silencing and enhancement of host defense, but some can be lethal as RNA silencing suppressor antagonists. Moreover, selected viruses produce lncRNAs by incomplete degradation of genomic RNAs. They do not replicate but may impact viral infection, gene regulation, and cellular functions. Finally, the host plant lncRNAs can also contribute during plant-virus interactions, inducing plant defense and the regulation of gene expression, often in conjunction with micro and/or circRNAs.

## 1. Introduction: lncRNAs

Over several decades numerous classes of noncoding RNAs (ncRNAs) have been identified in all organismal kingdoms. Among the three basic groups of ncRNA are short ncRNA, such as micro RNAs (miRNAs), small interfering RNAs (siRNAs), tRNA-derived stress-induced RNAs (tiRNAs), small nucleolar RNAs (snoRNAs) and piwi-interacting RNAs (piRNAs), middle size ncRNAs, and long non-coding RNAs (lncRNAs), >200 nt in length, such as cold assisted intronic noncoding RNA (COLDAIR), HOX antisense intergenic RNA (HOTAIR), etc. [1,2]. In particular, the genes encoding lncRNAs occur in very large numbers in the human genome (58,648 genes) and their regulative roles are implicated at all levels of gene expression, influencing multiple cellular activities. In general, lncRNAs account for 68% of ncRNAs [3,4].

In plant genomes, lncRNA transcripts situate as intergenic RNAs, antisense RNAs, intronic RNAs, and non-overlapping antisense RNAs [5]. LncRNAs can function at various levels including transcriptional, post-transcriptional, and translational regulation or even post-translational modification, having implications for dosage compensation, splicing, cell cycle, and differentiation, or for controlling diseases, and in turn play roles in flowering, morphogenesis, reproduction, crop yield or stress response. An example of regulation of flowering is lncRNA COLDAIR, which is downregulated by cold treatment, recruits a protein PRC2 which suppresses the expression of Flowering Locus C (FLC locus), and then induces flowering [6].

LncRNAs can regulate histone modifications at the chromatin level, via interacting with or acting as scaffolds for methylation and acetylation complexes. Such epigenetic effects are well described in the case of repressive complex PRC2 [7] or COMPASS-like complex [8], which are targeted by lncRNAs to the cognate chromatin sites and cause chromatin remodeling via histone methylation [9]. LncRNAs can also regulate DNA methylation via RNA-dependent DNA methylation process, interacting with DNA methyl transferases, or by gene silencing mechanisms [10].

At the transcription level, lncRNAs can act as co-factors that modulate the activity of transcription factors, e.g., during plant immune responses (expression of PR proteins [11,12]). LncRNAs regulate messenger RNA (mRNA) alternative splicing, can act as miRNA sponges or as precursors of miRNAs and siRNAs, or can mediate RNA decay and stability. Post-transcriptionally, lncRNAs participation in the regulation of translation and post-translational modification has been observed at various levels of protein synthesis. Overall, most of the nuclear lncRNAs do not function alone as independent factors but interact with other factors via complex (cascade) mechanisms, and there is no single universal function of lncRNAs [11].

**Figure 1:**
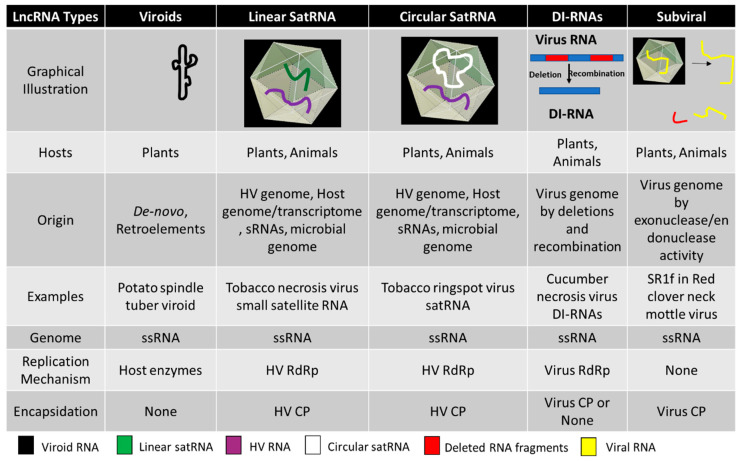
Schematic illustration of five types of subviral/virus-associated long non-coding RNAs (lncRNAs) related to plant pathogenesis and their basic attributes. ssRNA—single-stranded RNA, HV—Helper virus, RdRp—RNA dependent RNA polymerase, CP—Coat protein.

In this review, we focus on lncRNAs related to plant RNA viruses, both to those that do not originate from virus genomes (viroids and satellite RNAs) and those of viral origin (defective-interfering DI RNAs or subviral RNAs) (Figure 1). Various types of these viroids, satellite RNAs, and other virus-derived lncRNAs have been identified for many plant viruses that can modulate the overall viral infectivity and host transcriptome. Recent development in next generation sequencing (NGS) has made research on these lncRNAs cheaper and more extensive.

## 2. Viroids

Viroids are a group of infectious plant lncRNAs that are composed of RNA genomes and replicate by using the host enzymatic activities. Viroids carry the circular, single-stranded, non-coding RNA molecules that represent the smallest known replicons among all living objects, ranging between 246 to 401 nucleotides (nt) in length [13,14]. Viroid RNAs (vd-RNAs) do not carry any functional open reading frames and can be defined as circular lncRNAs.

Their mode of action along with the host processes makes the viroids unusually complex lncRNA molecules [15]. Uniquely, viroids can multiply and accumulate in the infected plant tissue by using host RNA polymerases. So far, no animal viroid RNAs have been described. Interestingly, circular single-stranded (ss) viroid RNAs replicate autonomously in plant cellular organelles, either in nuclei or in chloroplasts. No sequence homology with the host plant genome or with a potential helper virus genome has been observed among viroids. Currently, several thousand variants of viroid sequences are available in the National Centre for Biotechnology Information (NCBI) databank. Based on the presence of the central conserved region (CCR) containing the C-domain, viroids are classified into two families, the *Pospiviroidae* and the *Avsunviroidae*, each further divided into genera according to their RNA structures and relationships. A brief overview is given in Table 1 (see Supplementary Data, Table S1 for detailed elaboration) [16].

**Figure 2:**
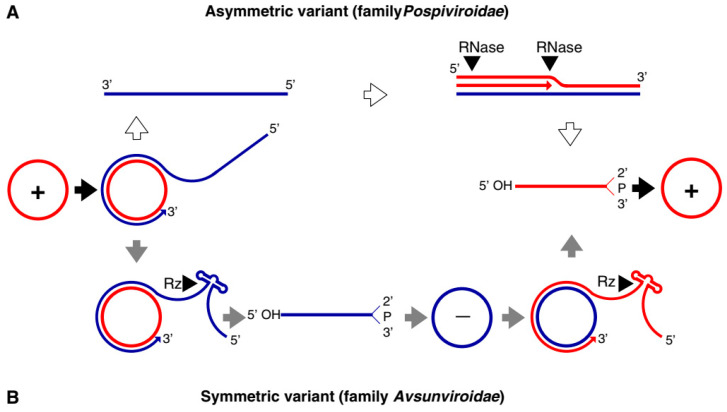
Rolling circle replication (RCR) in the *Pospiviroidae* and the *Avsunviroidae*. The members of *Pospiviroidae* replicate by the asymmetric RCR mechanism (**A**) inside the host nucleus with the help of the host enzymes. The circular + strand (Red) of viroid RNA is copied into the–strand (Blue) by the RCR, which is linearized by the host RNases, and the linear RNA is then used as the template for the synthesis of the + strand. Finally, the host ligases circularize the viroid RNAs. As shown in panel (**B**), the members of *Avsunviroidae* replicate by the symmetric rolling circle mechanism using the nuclear-encoded polymerase (NEP) in the chloroplast. Replication of RNA strands with both polarities are completed by the RCR mechanism. In this family, the viroid RNAs have autocatalytic ribozymes that catalyze the cleavage of the circular oligomeric form of the viroid RNAs into monomers (Diagram adapted from the Flores et al. FEBS Letters 567, 2004).

Both families of viroids adopt the rolling-circle replication (RCR) mechanism, but the *Pospiviridae* replicate by asymmetric and the *Avsunviridae* by symmetric rolling-circle mechanisms (Figure 2). *Pospiviroidae* replicates in the nucleus, where the host DNA dependent RNA polymerase II is involved in the transcription of the viroid RNA [17]. Studies of the PSTVd, the type species of the *Pospiviroidae,* have shown that the host transcription factor TFIIIA is required for the transcription of the viroid RNA. Out of two splicing variants, TFIII-9ZF and TFIII-7ZF, the latter is involved in the transcription of the viroid RNA, which is regulated by the ribosomal protein L5 (RPL5) [18,19,20]. In *Pospiviroidae* the monomeric circular viroid RNAs replicate by the RCR mechanism to produce an oligomeric longer-than-unit strand, that is cleaved into replicative monomeric RNAs by one or more host ribonucleases (RNases) most likely the members of RNase III family. The recognition of the cleavage site by host RNases is determined by the stem-loop structures in the viroid RNAs around the CCR region. Thus produced monomeric linear (ml) RNA serves as a template for the synthesis of the ml (+) strand RNA [21]. The ml (+) RNA is then ligated into the circular (+) RNA. It has been demonstrated by Nohales et al. (2012) that the viroids force host DNA-ligase, most likely DNA-ligase I, to act as RNA ligase to circularize the ml (+) strand RNA [22,23].

The members of *Avsunviroidae* replicate in the chloroplasts by the symmetric RCR mechanism, where (+) stranded circular viroid RNAs are copied into the oligomeric (-) RNAs that are cleaved and ligated to the mc (-) strands. The mc (-) strands are used as a template to undergo a second round of the RCR to produce mc (+) RNA [17]. The nuclear-encoded polymerase (NEP) are predicted to catalyze the transcription of the viroid RNA at a specific initiation site, which is located at the (A+U)-rich terminal loops in the avocado sun blotch viroid (ASBVd), and a 6–7 bp GUC-rich double-stranded RNA motif in the peach latent mosaic viroid (PLMVd) [24,25,26,27]. The cleavage of the replication intermediates of both the polarities required for the replication is known to be facilitated by the autocatalytic hammerhead ribozymes (HHR) in *Avsunviroidae* [28,29]. Besides, the HHR can also catalyze the ligation reaction in-vitro to circularize the ml RNAs. However, the efficiency of the HHR and the requirement for a higher concentration of Mg^2+^ beyond physiological conditions questioned the feasibility of the reaction in vivo [21]. However, Nohales et al. (2012) have shown that in the replication of eggplant latent viroid (ELVd) the catalytic activity of the chloroplastic isoform of eggplant tRNA ligase mediates the circularization of both ml (+) and (-) strands. Another work also demonstrated that a recombinant eggplant tRNA ligase can mediate circularization in ASBVd, PLMVd, and Chrysanthemum chlorotic mottle viroid (CChMVd) [30].

The extensive secondary structure plays a critical role in the viroid life cycle, such as in host plant invasion, replication, pathogenesis, and transport. Viroid RNA structures have been studied in great detail by using SHAPE analysis whereas their structural 3D complexity has been confirmed via the direct visualization of single-RNA molecules by atomic force microscopy [31,32]. Structure prediction of the stability of viroid molecules can help explain how viroids escape the RNA silencing pathways [33,34].

Viroid infections often cause disease symptoms in such important crops as apple, avocado, coconut, grapevine, hop, peach, potato, tomato, and others [35]. Recent findings shed new light on molecular mechanisms of interaction, securing regulation of viroid replication in the plant cell. For example, potato spindle tuber viroid (PSTVd), a model viroid, requires a splicing form of transcription factor IIIA (TFIIIA-7ZF) for its multiplication via direct interaction with a splicing regulator RPL5, which in turn favors the expression of TFIIIA-7ZF [18]. There is some evidence that viroid replication links to RNA silencing, e.g., for PSTVd that can replicate efficiently in the presence of DCL4, but not of DCL2 host genes [36]. Interestingly, small RNAs originated from PLMVd also appeared to participate in the cleavage of chloroplast mRNA for heat shock protein [33], likely inducing the pathogenic symptoms of viroid infection. Similarly, the miRNA-induced cleavage of the virulence region of PSTVd targeted the pyrophosphatase mRNA. PSTVd also targeted a bromo domain-carrying protein VIRP1 in tomato [37]. PSTVd failed to infect VIRP1-suppressed *N. benthamiana* plants, signifying that this viroid should be viewed as a functional lncRNA [38].

Viroids have emerged as productive tools with which to study the interactions not only of their replicable lncRNAs, but also the plethora of functions of host lncRNAs in general [39]. Current research focuses on the transcriptomic analysis of viroid-infected plants to identify e.g., the patterns of viroid-siRNA-induced RNA silencing of host mRNAs. This helps to obtain a holistic picture of viroid-induced regulation or the widespread degradation of the host gene expression. Yet translation also appears to be affected/regulated by viroids [40]. It has been shown that viroid molecules were present in the ribosomal fractions [41]. In general, the above and similar findings provide new insights to better understand viroid biology and thus the means for viroid control.

Among several hypotheses regarding the origination of viroids, Kiefer et al. (1983) suggested that they might come from transposons or retroviruses [42]. However, more currently, because they contain catalytic RNA elements, Flores et al. (2014) considered viroids as remnants of ‘the RNA world’ that arose earlier than DNA and proteins. In some retrotransposons ribozyme activities, were found, further emphasizing a link between TEs and catalytic RNAs [43]. This is supported further by recent findings with ASVd, another model viroid, which has been shown to bear a double hammerhead ribozyme, also found in mobile elements and other viroid-like RNAs [44,45]. Recently, Catalan et al. (2019) have proposed a possibility of the *de novo* origin of viroid-like replicons via a parsimonious scenario. From the pool of various RNAs in eukaryotic cells, some can circularize and serve as seeds of the process [46].

NGS has been widely used in viroid research for the discovery of new viroids and diagnostic purposes. The technology has been employed to analyze viroid sequences and host gene expression (transcriptomic) in response to viroid infection [47]. Progressive filtering of overlapping small RNAs-1/-2 (PROF1/PROF2) uses deeply sequenced small RNAs and assembles them into circular RNAs representing possible viroids and satellite RNAs. This software discovered the apple hammerhead viroid (AHVd) from apple plants and a novel grapevine latent viroid (GLVd) from an old grapevine plant [48]. Recently, the PROF2 in combination with the assembly software Velvet allowed the discovery of viroid-derived small RNAs responsible for host RNA silencing in *Coleus blumei* infected with coleus blumei viroids (CbVds). Based on the NGS data it has been revealed that the central conserved region (CCR) of the viroid is pivotal in the biogenesis of sRNAs for both host RNA silencing and the genome replication of the viroids [49]. Deep sequencing revealed the viroid heterogeneity, with 3939 variants of inoculated parent PLMVd detected after six months of infection in the natural host [50]. Similarly, transcriptome analysis of the citrus bark cracking viroid (CBCVd) revealed a new variant of the viroid from citrus plants in Pakistan. Interestingly, the viroid was highly diverse phylogenetically from CBCVd found in other Asian countries [51]. Therefore, NGS has proved to be an important asset in the discovery of novel viroids and can be used for viroid screening and quarantine.

## 3. Replicative lncRNAs: Defective and Defective-Interfering RNAs

Defective (D) and defective interfering (DI) RNAs represent of the class of lncRNAs that are derived from the helper RNA virus genome by one or more premature termination and reinitiation events, resulting in functional replicative templates [52]. The replication of DI RNAs is completely dependent on the helper virus, which usually limits virus replication and alleviates symptoms. Sometimes, however, the DI RNAs can intensify the disease symptoms, e.g., as observed for broad bean mottle virus (BBMV) [53,54]. In some cases, the virally-derived RNAs do not interfere with virus disease, and these are referred to as defective (D) RNAs. In this review, if not further specified, we will use the inclusive term defective RNAs for both D and DI RNAs. The majority of defective RNAs are noncoding [55,56,57,58]. Defective RNAs could accrue up to 60% of viral RNA during infection, but the encapsidated fraction might be much lower. Interestingly, defective RNAs are often not vector-transmissible [52,59,60]. The systemic movement of these RNAs seems to be host dependent, e.g., the cucumber mosaic virus (CMV) DI RNA moves systemically in tobacco species but not in tomato or cucurbits [60,61,62]. The genetic relatedness to the helper virus distinguishes defective RNAs from satellite RNAs, as the latter are also dependent on a helper virus but genetically (sequence-wise) are not related.

Current analytical developments, especially in NGS sequencing, have progressed rapid advancements in the understanding of the mechanisms of the generation of DI RNAs, and their role as signals promoting antiviral immunity during infection, affecting viral dynamics and evolution during host-virus interactions. The diversity of defective RNAs is much larger than initially detected and they appear to be present in virtually every virus population. The use of single-cell sequencing technology and bioinformatics detected jumbled or rearranged viral sequences, and their populations are not the same if generated in different hosts [63]. Truncated viral genomes result from large internal deletions that remove several or all essential genes but retain replication and/or packaging signals. Yet other species result from multiple recombination events and various sequence rearrangements [64].

**Figure 3:**
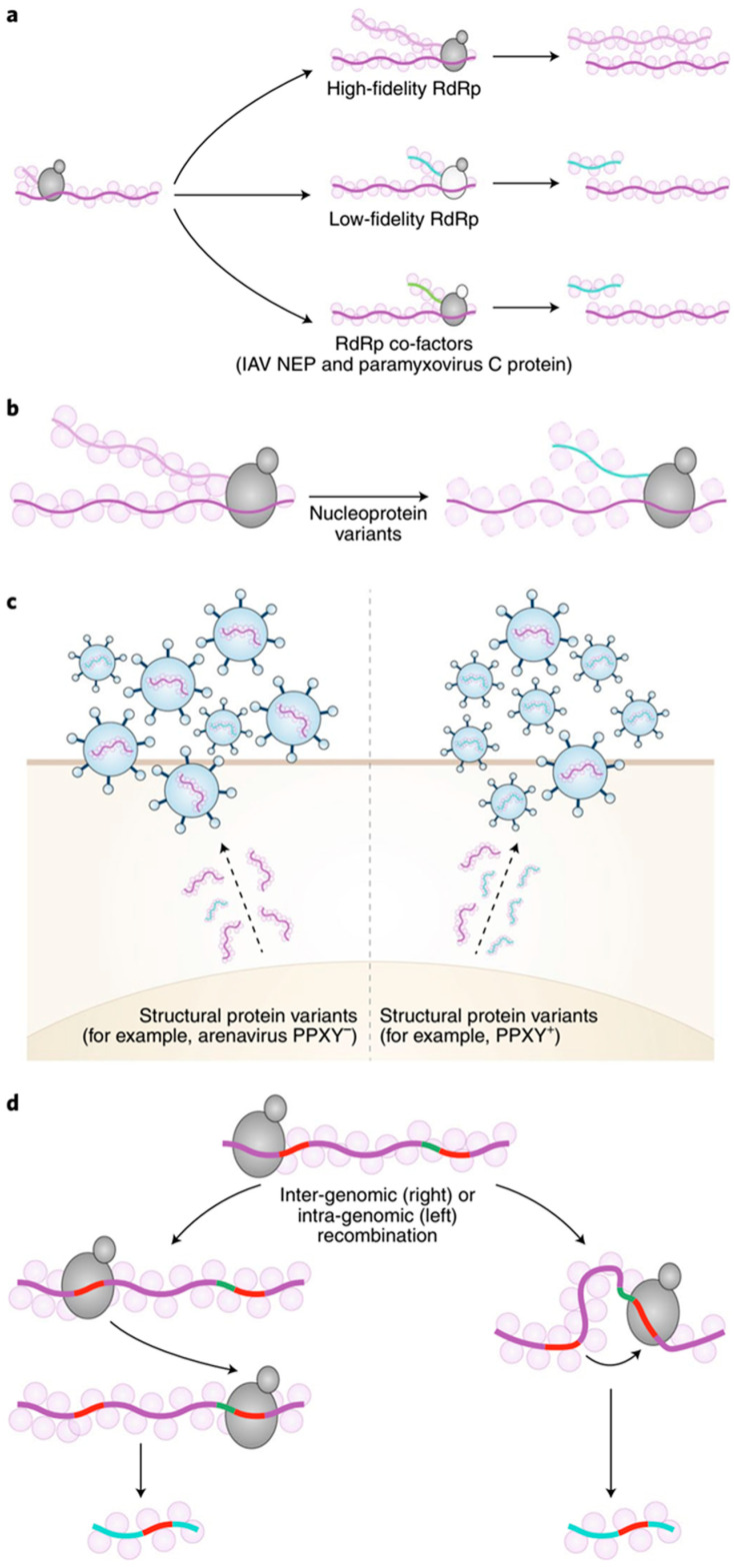
The schematic representation of mechanisms of the generation of defective viral RNAs. (**a**) Either altered RdRp fidelity due to mutations or effects of virus-encoded co-factors, such as the influenza A virus (IAV) NEP or the paramyxovirus C protein, can favor the generation of D RNAs. (**b**) Variants of the nucleoprotein with altered binding to viral RNA can promote D RNA generation. (**c**) Altered structural proteins, such as the PPXY domain in the matrix protein of arenaviruses, can lead to encapsidation of the D RNAs. (**d**) Inter- and intra-recombination events using homologous sequences (red) can lead to the formation of D RNAs. (Courtesy Vignuzzi and Lopez, 2019, *Nat. Micro*.)

Defective RNAs generally consist of the non-contiguous portions of the helper virus genome, synthesized by the viral RNA-dependent RNA polymerase (RdRp). The replication-dependent template-switching mechanism, either the replicase dependent or the forced template-switching mechanism, are among the well documented and popular means for synthesis of the defective RNAs [65]. The analyses of species such as *Cucumber necrosis virus*, *Brome mosaic virus*, *Turnip crinkle virus*, *Cucumber mosaic virus*, *Bovine viral diarrhea virus*, and *Hepatitis C virus* have shown that RNA breaks, strong hairpin structures, or that AU–rich stretches enhance the replicase in order to switch the template between the donor RNA and the acceptor RNA, leading to mosaic or truncated progeny RNAs, with loss in peptide coding functionality, but retaining the ability to replicate [66,67,68,69,70]. Besides these extensive recombination events, other events like point mutations, hypermutations, frameshifts, single nucleotide deletions, and copy backs are known to synthesize the various classes of defective RNAs. Copy backs involve sequence rearrangements resulting in reverse complement duplications, forming stem-like structures. Defective RNAs can also be generated *de novo* randomly due to faulty viral proteins such as mutated RdRp, nucleoprotein variants, and other structural proteins that affect replication and recombination, as illustrated in Figure 3 [63].

To date, DI RNAs resulting from the above mechanisms have been characterized for a variety of plant RNA viruses carrying positive-sense, negative sense, double-stranded and ambisense genomes [52,60]. For the members of *Crinivirus*, DI RNAs comprise a mosaic of the multipartite parental viral genome [71], while deletions are characteristic of DI RNAs in *Tombusviruses* [65,72]. Tobra-, Potex-, Furo-, Peclu-, and Beny-viruses accumulate single-deletion defective RNAs. Although DI RNAs derived from the same virus can vary in their structure, some elements are highly conserved, namely those essential for RNA viability, such as retaining the cis-acting sequences essential for RNA replication by viral RdRp or encapsidation signals.

The roles of viral replicase proteins, the replication/recombination sequence domains, host factors, and growth conditions have been found to affect the above mechanisms of defective RNA generation [73]. The role of replicase proteins in RNA recombination has been shown by using the mutant of the BMV helicase-like 1a protein mutant, where the mutant affected the nature of the RNA recombination products in comparison to the wild type (wt) 1a protein [74]. Similar effects have been demonstrated in many animal viruses where recombinant RdRp are found to be associated with attenuated virus accumulation, in most cases correlated with the accumulation of the defective RNAs [75,76,77,78]. Besides RdRp, other structural proteins as shown in Figure 3, are also known to be involved in the generation of defective RNAs, like the nuclear export protein (NEP) in the influenza virus which increased DI production, the paramyxovirus protein C increases the DI RNAs via the copy-backs [79,80]. Similarly, in *Potato mop-top virus* the 8K protein and the triple gene block 1 (TGB1) proteins are known to alter the biogenesis of the DI RNAs [81]. The conserved sequence domains (elements) or secondary structures in the viral RNAs are also known to control the mechanism of replication and recombination, contributing directly to the generation of defective RNAs. For example, the enrichment of the AU sequences in the RNA2 and RNA3 of the BMV is known to increase the frequency of imprecise crossovers [82,83,84].

Host factors can influence the mechanism of the DI-RNA generation. Using the yeast single gene knock out library, several dozens of host genes that could affect virus RNA recombination have been identified in tomato bushy stunt virus (TBSV). The host exoribonuclease-like *XRN1* is found to promote the formation of DI-RNAs [65,85]. The role of RNA editing causing D RNA diversity has been shown for human RNA viruses [86,87]. Thus, the current data suggest a combination of such factors, including random errors such as deletion formation by viral polymerase due to the lack of proofreading activity, and mutations in the other structural proteins, as contributing to the biogenesis of defective RNAs. Besides, the generation of copy-back sequences appears to be another factor, which is not completely random, being directed by specific signal sequences [88].

The pathogenesis of the DI RNAs in *Tombusviruses* and *Cucumoviruses* has been extensively studied [52,65] and *de novo* generation of DI RNAs has been observed for the first time in *Tombusviruses*, after serial passages of DI RNA-free viral RNA through host plants. The presence of a DI RNA considerably attenuates *Tombusvirus* infection, from lethal necrosis to persistent symptoms. In general, DI-RNAs have complex relationships with the helper virus (HV) regarding the competition for viral- and host factors, and mitigation of antiviral responses and/or disease symptoms in the host plant. D RNAs have also been reported in the tomato black ring virus (TBRV), one of the *Nepoviruses*. The D RNA formed by a single deletion in the RNA1 molecule interfered with the replication of TBRV. Recently, DI-RNA has also been shown to increase the vertical seed transmission of the virus [89,90].

Defective RNA-virus interactions evolve into mutually beneficial combinations. Such domestication processes enhance virus fitness, giving the helper virus competitive advantages in its relationship with the host, mostly by moderating disease symptoms [65]. The rate of evolution of DI RNAs appears to be faster than that of the helper viruses due to their small size and reduced competition for translation. However, the shorter genome of DI RNAs has disadvantages for cooperative binding to viral movement proteins, reducing their cell-to-cell movement in the infected plants. An example of the interplay between virus and molecular DI RNA parasite has been demonstrated for *Potato mop-top pomovirus*, where regulation of virus accumulation depended on the antagonistic roles of the relative levels of 8K protein and D RNAs (the 8K gene is needed for efficient virus accumulation but D RNA impairs efficient virus accumulation) [81].

The mechanism of interference is not clearly understood but the possibilities include (i) competition for transacting factors, (ii) interaction with viral products, (iii) inhibition of pathogenicity determinants, and/or (iv) activation of RNA interference [81]. The first hypothesis, however, is not able to explain the persistence of infection with *Tombusviruses* [72,91]. Functional interactions between genomic and DI RNAs are also not directly responsible for attenuation of symptoms, at least for some DI RNAs [92]. As for the third hypothesis, the data revealed efficient induction of siRNAs by DI RNAs but DI RNAs were poor targets for virally derived siRNAs [59]. This suggests that DI RNAs contribute to efficient degradation of the helper virus genome, via inducing systemic RNA interference ahead of virus spread.

Recent NGS data for animal RNA viruses demonstrate that hundreds of defective RNAs can arise within a single viral infection, but only some are repeatedly detected in different samples [88]. More research is required towards understanding whether similar processes occur for plant RNA viruses, but also into how plant viruses and their DI RNAs co-evolve within the framework of the host and the outside environment [63].

## 4. Replicative RNAs: Satellite RNAs

The lack of sequence homology to the helper virus distinguishes defective RNAs from sequence-unrelated satellite (sat) RNAs, although they are also dependent on the helper virus (Table S2). In contrast to viroids, sat RNAs both replicate and encapsidate by a helper virus [93]. These molecular parasites of viruses usually affect (attenuate or enhance) viral symptoms [94,95]. The first example of pathogenic satRNA was reported in 1977, causing lethal necrosis disease in tomato plants by CMV [96]. Some results suggest similarities between satRNAs and viroids, e.g., based on the fact that satRNA of CMV replicated in the nucleus independently of the helper virus [97]. 

As far as their general organization, the satRNAs include three categories of single-stranded (ss) RNAs: large linear ss RNAs (0.7–1.5 kb) that encode a protein, small linear ss RNAs (less than 0.7 kb), and small circular ss RNAs. An example of the first category is *Bamboo mosaic virus* satRNA, which encodes an RNA-binding protein [98]; however, as such, it cannot be considered as a lncRNA. The small satRNAs, both linear and circular, do not encode any protein, a feature of classical lncRNAs. SatRNAs associated with different helper viruses belonging to various families and genera are briefly tabulated below in Table 2. (See Supplementary data in Table S2 for more elaborate detail). Some examples of small linear satRNAs are tobacco necrosis virus small satellite RNA, tomato bushy stunt virus satellite RNA, peanut stunt virus satellite RNA, tobacco bushy top virus satellite RNA, and others [99]. This well-studied category of satRNAs is very analogous to other lncRNAs, especially in the case of CMV satRNAs [100,101]. The majority of CMV satRNAs do attenuate CMV infection but some can induce lethal infection, leading to necrosis or chlorosis due to the presence of specific sequences or silencing of some host genes [102]. The systemic necrosis on tomato is likely caused by triggering programmed cell death with CMV D-satRNA [103], whereas CMV Y-satRNA-derived siRNAs are known to saturate the virus-encoded suppressor of RNA silencing (VSR), leading to attenuation of the symptoms [104]. In *Nicotiana tabacum,* a large amount of siRNAs derived from Y-sat RNA shows yellowing of leaves caused by the down-regulation of chlorophyll biosynthetic gene *ChlI* by RNA silencing [95,105]. The peanut stunt virus (PSV) showed exacerbated and accelerated symptoms in the presence of the PSV satRNAs. The *Nicotiana benthamiana* plants co-infected with the satRNAs showed a higher fold change of differentially expressed genes (DEGs) involved in the biosynthesis of secondary metabolites, translation, ribosome biogenesis, and RNA metabolic processes [106].

Zhu et al. (2011) suggest that satRNA attacks CMV RNA via RNA silencing [107], leading to enhancement of host defense response, e.g., by guiding RNA-directed DNA methylation [108]. Since RNA silencing plays an important role in defense against RNA viruses, this has been surmised as the cause of the origination of replicable sat RNAs, because these could strengthen the silencing activity via the RDR6-dependent siRNA amplification system [108,109].

Interestingly, the third category of small circular satRNAs is called virusoids, because they are similar to viroids but are dependent upon the helper virus [110]. The circular satRNAs have the ribozyme for autocatalytic cleavage and ligation required for their replication by rolling circle mechanism [111]. These satRNAs can be encapsidated either as circular RNAs or linear RNAs; the linear forms circularize in plant cells during replication [112] Well studied examples of this group include tobacco ringspot virus (TRSV) satRNA, which has a ribozyme activity [113], and rice yellow mottle virus (RYMV) satRNA that encodes a possible highly basic, active peptide [114].

The structures of satRNAs are known to be conserved and are required for successful replication. For example, in lucerne transient streak virus satellite RNA (LTSV satRNA), most of the mutations reverted to the wild type by 21 days post-inoculation (dpi) and had wild-type secondary structures [111]. Thus, satRNAs have a very interesting relationship with the helper virus (HV) and the host. Some satRNAs are pathogenic to the HV, which means they attenuate both HV accumulation and symptom induction [115], whereas other satRNAs attenuate HV accumulation but disease symptoms are aggravated in the host plants [116]. SatRNAs can also act as the RNA silencing suppressor (RSS) antagonist to aggravate symptoms in the host plants [104]. The relationship between satRNAs and HV, along with the host plants, can be exploited for therapeutic use for the development of virus-resistant crops [117,118,119]. Moreover, studies of the satRNAs associated with animal viruses related to human diseases are also recently progressing, such as Hepadnavirus associated satellite-like RNAs (HDV), and Adeno associated satellite virus (ASV) [120].

## 5. Nonreplicative Lnc RNAs of Plant Viruses

Plant RNA viruses generate subviral RNAs (subgenomic RNAs, sgRNAs) for high-yield translation of downstream ORFs. sgRNAs can be produced by internal initiation from the subgenomic promoter, by premature termination, leader primed transcription, or discontinuous transcription. BMV, one of the most commonly studied plant RNA viruses, transcribes sgRNA4 from an internal subgenomic promoter in the RNA3, which is translated into the coat protein [121]. By the same token, BMV RNA3 transcribes its 5′ portion by premature termination producing sgRNA3a to facilitate the translation of virus movement protein [122].

However, selected viruses produce non-replicating RNAs that do not encode any protein, often by incomplete degradation of genomic RNAs. Such degradation can be due, e.g., to cellular 5′ to 3′ XRN1 exonuclease, until it stops at a structured element. Such lncRNA products may then interact with different cellular factors used for transcription, translation, splicing, or RNA interference [123,124,125]. One example of lncRNA degradation product has been found in red clover necrotic mosaic tombusvirus (RCNMV). This 400 nt lncRNA, dubbed SRf1, is a 3′ UTR derivative of RNA1, gets encapsidated, and functions as a repressor of synthesis of the negative-strand of RCNMV RNA [126]. Yet, in *Benyviridae*, the beet necrotic yellow vein virus generates a non-encapsidated 500 nt lncRNA (RNA3sub) of yet to be established function [127].

Among common features shared by virally-derived lncRNAs are their highly stable structures, the use of RNAPII or RNA degradation mechanisms for their production, their importance in the infection cycle, and their direct intervention in gene regulation. This can modulate the hormonal and metabolic signaling pathways to enhance viral infection. For example, the citrus tristeza virus (CTV) produces a lncRNA called low molecular weight tristeza 1 (LMT1), which is associated with maintaining virus accumulation, movement, and infectivity by lowering the production of salicylic acid (SA) and reactive oxygen species (ROS) required for antiviral defense [128]. Thus, viral lncRNAs play important roles in disease development [129], and they can be targeted by RNAi mechanisms, possibly to overflow the cellular defense system (likely in the form of counteracting competing-endogenous ceRNAs) [130].

## 6. Host lncRNAs during Plant-Virus Interactions

Regarding the origination of plant lncRNAs, one theory links their biogenesis to TE elements [131,132]. Because TEs closely link to retroviruses, they are probably derived from a common ancestor [133]; lncRNAs might have evolved from viruses through TEs, but in such a way that they resisted/survived RNA silencing [134]. Along these lines, computer studies on human DNA viruses have revealed homologies to human lncRNAs, suggesting an evolutionary association between lncRNAs and viruses. Consequently, viruses, especially cancer-related viruses, may have evolved from noncoding RNA transcripts [135].

Studies have been conducted to evidence the contribution of host lncRNAs during plant-pathogen interactions [136]. For instance, lncRNAs appear to function in plant defense against viral infection [137]. In the TYLCV (tomato yellow leaf curve virus)-resistant line of tomato, different regulation profiles of lncRNAs, and long noncoding natural antisense transcripts (lncNATs), were observed, as compared to susceptible lines; similar effects were detected in *N. benthamiana* [138]. Host lncRNAs had participated in defense signaling, controlling the expression of miRNA, where the transcription of miRNA-target genes was upregulated by lncRNAs.

In another work on the TYLCV-tomato system aiming at the identification of lncRNAs as key regulators of gene expression, the NGS RNA-seq revealed different patterns of lncRNAs and circRNAs in infected vs. uninfected susceptible plants [139]. The silencing (by VICS) of the circ RNA’s parental genes resulted in decreased TYLCV virus accumulation. The circRNAs are among the novel endogenous lncRNA in eukaryotes associated with many biological functions such as development, and biotic/abiotic stress. The circRNAs are generated during post-transcriptional processing via the back splicing of precursor mRNAs [140,141,142]. The authors identified novel lncRNAs and circRNAs that functioned as regulators of TYLCV infection, several acting as susceptibility genes in TYLCV infection, whereas some exonic circ RNAs upregulated the expression of host genes. Similarly, in response to the maize Iranian mosaic virus (MIMV), the maize plants showed differential expression of the circRNAs such that 155 circ RNAs were upregulated whereas five were down-regulated. Interestingly, the secondary structure analysis of the differentially expressed circRNAs predicted that 33 of those RNAs might interact with 23 maize miRNAs that are responsible for the regulation of plant metabolism and development [143]. In the watermelon plants, the cucumber green mottle mosaic virus (CGMMV) infection differentially expressed 67 lncRNAs and 548 circRNAs that are responsible for the metabolic pathways, such as phenylalanine metabolism, citrate cycle, and endocytosis [144]. The role of circ- and other lnc-RNAs in up- and down-regulation of expression of their parental genes have been also observed in rice [145] and kiwifruit [146]. The circ RNAs can also act as miRNA sponges to prevent the degradation of target mRNAs [147,148,149]. All these examples illustrate that host lncRNAs play a complex role in the regulation of defense responsive genes, observed in plants not only for viruses but for other pathogens too [136].

## 7. Final Remarks

Several decades of extensive research on viroids have contributed significant progress toward the understanding of interactions of vd-RNAs with plant cell components, especially to explain the involvement of vd-sRNA in viroid-induced disease symptoms and induced gene expression, and the role of RNA silencing. However, further analyses are required on the mechanisms of viroid-induced host gene expression, as well as on the contribution of circRNAs in the regulation of translational machinery. Structure prediction techniques will further the study of viroid biology, especially concerning means of escape from RNA silencing surveillance.

Likewise for D RNAs, recent NGS sequencing projects have revealed considerable diversity among D RNAs in plant viruses. Nevertheless, some questions remain, including the detailed molecular mechanisms of the D RNA biogenesis, their control of host-virus pathogenesis, the degree to which D RNA populations contribute to virus evolution and host range, and how the interactions of D RNAs with viral and/or host factors affect the stages of the virus life cycle or induction of symptoms.

As for host plant lnc RNAs and plant responses to pathogens/viruses, although a great number of host- and pathogen-related lncRNA has been discovered in many organisms, and considerable efforts have been undertaken to recognize lncRNA-mediated plant defense against viruses, the precise antipathogenic defense remains largely obscure, e.g., in terms of the link to the RNA silencing machinery. In general, lncRNAs have been found to play a role in the modification of chromatin architecture, and to interact with transcription and translation factors, with proteins of signaling cascades, or with other cell host factors. These lncRNAs could likely act to overthrow cellular defense systems. Understanding the relevance of lncRNAs to disease in terms of virulence development and anti-viral immunity will change our views on RNA regulations and will facilitate the designing of new antiviral strategies.

## Figures and Tables

**Table 1:** A brief overview of the various members of viroids belonging to *Avsunviroidae* and *Pospiviroidae*. (Elaborated details on all the members reported in the International Committee for Taxonomy of Viruses (ICTV) and references are provided with Table S1 in the Supplementary Data).

Viroid Classification	Type Species	Genome and Pathogenesis
Avsunviroidae		
Avsunviroid	Avocado Sun Blotch Viroid (ASBvd)	The viroids in this family have a circular genome of 247–399 nucleotides. Viroids are characterized by a specific central conserved region (CCR) in the RNA and have hammerhead ribozymes (HHR) required for symmetric rolling circle replication in the chloroplast. Most viroids are symptomatic to the host whereas some can be asymptomatic.
Pelamoviroid	Peach latent mosaic viroid (PLMvd)	
Elaviroid	Eggplant latent viroid (ELVd)	
Pospiviroidae		
Pospiviroid	Potato spindle tuber viroid (PSTVd)	The genome size ranges from 246 to 371 nucleotides. The viroids lack central conserved region (CCR) and ribozyme activity. Replication in the nucleus by asymmetric rolling circle replication is catalyzed completely by host enzymes. It can infect a wide range of hosts including *Solanaceae*, *Asteraceae*, *Compsitae*, and others including various economically important fruit crops like apples, citruses and some ornamental plants.
Hostuviroid	Hop stunt viroid (HSV)	
Cocadviroid	Coconut cadang-cadang viroid (CCCVd)	
Apscaviroid	Apple scar skin viroid (ASSVd)	
Coleviroid	Coleus blumei viroid (CBVd)	

**Table 2:** A brief overview of various linear and circular satRNAs showing respective belonging to different families and genera of viruses. (More elaborate details can be found in Table S2, provided with the Supplementary data).

Family/Genus of Helper Virus	Example	Genome and Pathogenesis
**Linear Small Satellite RNA**		
Tombusviridae	Tomato bushy stunt virus (TBSV) satellite RNA	The genome is linear long non-coding RNA, ranging from 339 to 901 nucleotides. Most satRNAs are pathogenic to the helper viruses whereas some, like black beet scorch virus (BBSV) satellite RNA, is known to intensify symptoms and virus accumulation.
Bromoviridae	Cucumber mosaic virus (CMV) satellite RNA	
Umbravirus	Groundnut rosette virus (GRV) satellite RNA	
**Circular Sat RNAs**		
Secoviridae	Tobacco ringspot virus (TRSV) satellite RNA	Also known as virusoids, they have circular long non-coding RNAs ranging from 220 to 457 nucleotides. The RYMV satRNA is the smallest known pathogenic subviral agent. The RNA secondary structure is conserved with a hammerhead ribozyme. They replicate by rolling circle mechanism using the helper virus machinery and are encapsidated by the helper virus coat protein. Mostly pathogenic to the helper virus, leading to attenuation of the symptoms in host plants. The replication of satRNAs is known to be supported by heterologous helper viruses across different species.
Luteoviridae	Barley yellow dwarf virus (BYDV)	
Sobemovirus	Rice yellow mottle virus (RYMV) satellite	

## Supplementary Materials

The following are available online at https://www.mdpi.com/2076-0817/9/9/765/s1, Table S1: The list of viroid published by the International Committee on Taxonomy of Viruses (ICTV) and a brief description of the characteristics of the members of Avsunviroidae and Pospiviroidae. The table lists only the species that has NCBI Genebank accessions. *—Possible members, TS—Type species, RNP—Ribonucleoprotein., Table S2: The list of linear and circular satRNAs, and a brief description of the relationship of satRNAs with helper virus and the plant host. *—Possible members, satRNA—Satellite RNA, DI-RNA—Defective Interfering RNA, CP-ORF—Coat protein-open reading frame.
