# Supplementary material for: Long Noncoding RNAs in Plant Viroids and Viruses: A Review
Source: Pathogens. 2020 Sep 18;9(9):765. doi: 10.3390/pathogens9090765 (PMC7559573; doi:10.3390/pathogens9090765)
Supplement: Supplementary file 1 [file pathogens-09-00765-s001.pdf]

Table S1. The list of viroid published by the International Committee on Taxonomy of Viruses (ICTV) and a brief description of the characteristics of the members of *Avsunviroidae* and *Pospiviroidae*. The table lists only the species that has NCBI Genebank accessions. * - Possible members, TS – Type species, RNP – Ribo-nucleoprotein.

**Genus**	**Viroid**	**Genome size (bp)**	**Remarks**	**Ref**
		Family -*Avsunviroidae*		
Avsunviroid	Avocado Sun Blotch Viroid (ASBvd)^{TS}	247	Avocado is the primary host. Causes symptoms on fruits like sunken crevices of white, yellow, or reddish color, and can be necrotic in severe infection	[1]
Pelamoviroid	Chrysanthemum chlorotic mottle viroid (CCMvd)	399	Only found in Chrysanthemum. It Causes Yellow-green mottling, chlorosis, and dwarfed size in infected plants.	[2]
	Peach latent mosaic viroid (PLMvd)^{TS} – peach, calico	337, 349	Can infect wide range of temperate fruits. May be symptomless or causes yellow, chlorotic mosaics and delays in foliation, flowering, and ripening, with fruit deformation and discoloration	[3]
Elaviroid	Eggplant latent viroid (ELvd)^{TS}	335	Only infects eggplants and are symptomless. The viroid has been widely used as model system for molecular studies and recombinant protein expression.	[4]
		**Family *Pospiviroidae***		
Pospiviroid	Chrysanthemum stunt viroid (CSVd)	356	Wide host range including Asteraceae and Solanaceae. Cause light green young leaves, chlorotic spots, stunting, small leaves and flowers, and decreased rooting ability	[2]
	Citrus exocortis viroid – citrus, tomato (CEVd – cit, CEVd – tom)	371, 372	Infects tomatoes, citrus and some ornamental plants. In tomatoes, it causes severe effects by imposing ribosomal stress. In citrus, it shows leaf epinasty, stunting, and necrosis of the leaf midvein.	[5], [6]
	Columnea latent viroid(CLVd)	370	Limited host range with an asymptomatic infection in some ornamental plants but necrosis on leaf veins, plant stunting and yield reduction found in Solanaceae species. Asteraceae species and cucumber are among other hosts. The terminal right domain of the virus genome is the 'host adaptation region'.	[7], [8]
	Iresine viroid 1 ((IrVd-1-ire)	370	It is asymptomatic. Found only in some ornamental plants.	[9]
	Mexican papita viroid (MPVd)	360	It causes serious disease outbreaks on greenhouse tomato in North America. First isolated from *Solanum cardiophyllum* (Mexican papita), causes symptoms of epinasty, stunning and veinal necrosis.	[10]

	Potato spindle tuber viroid (PSTVd)^{TS} – potato, intermediate, tomato	348, 356, 359	It is type member of the *Popsiviridae.* Infects several members of *Solanaceae*, and few others like avocado and sweet potato. It causes leaf curling, stunted growth, epinasty and venial necrosis. It is a well-studied viroid, known to silence various genes in the host like, *StTCP23*, *CalS11-like, CalS12-like*, chloride channel protein *CLC-b-like, RPS3a-like mRNA* etc.	[11], [12], [13], [14]
	Tomato apical stunt viroid – tomato (TASVd)	360	One of the most prevalent *Popsiviridae,* in ornamental plants in Netherlands. Mostly infects members of Compositae and Solanaceae families. Causative agent for the 'Rasta Disease' in tomato, symptomized by stunting; epinasty, crumpling, and chlorosis of leaves; and necrosis of leaf veins, petioles, and stems.	[15], [16], [17]
	Tomato chlorotic dwarf viroid (TCDVd)	360	Tomato is the primary host, also infects other plants, mostly asymptomatic. An infected tomato plant shows chlorosis of leaves and dwarfing.	[18]
	Tomato planta macho viroid – tomato (TPMVd)	360	TPMVd leads to severe stunting, strong epinasty in leaves, and decreased fruit in tomato plants. One of the viroids that can be horizontally transferred from pollen to the mother plant. Terminal left (TL) domain is the determinant for the phenomenon.	[19],
Hostuviroid	Hop stunt viroid (HSV)^{TS} – hop, citrus, cucumber, peach, plum	297, 299, 303, 297, 297	Infects a wide range of woody plants like grapevine, citrus, plum, peach, fig, mulberry, pear, pistachio and almond. HSvd translocate in the host plant as an RNP complex with host phloem protein 2 in cucumber (CsPP2).	[20], [21]
Cocadviroid	Citrus bark cracking viroid - citrus (Citrus viroid IV)	284	It infects mainly citrus and citrus relatives, and other herbaceous plants like herbaceous hosts such as cucumber, tomato, eggplant, datura, chrysanthemum, and gynura. It is a major pathogen in hops, causing a huge decline in the yield. Symptoms in hops include plant stunting, leaf yellowing and down curling, small cone formation and dry root rot.	[22], [23]
	Coconut cadang-cadang viroid (CCCVd)^{TS} - coconut palm, oil palm	246, 297	It is a lethal disease characterized by orange spotting symptoms in palm trees. The disease known to progress in three different stages. The overall time of appearance of the first symptoms to death of the plant is from 8-16 years.	[24], [25]
	Coconut tinangaja viroid (CTiVd) - coconut palm	254	It has 64% sequence identity with the CCCVd and they differ only in symptomatology. The disease has been reported only in Guam.	[26]
	Hop latent viroid (HpLVd) – hop	256	Majorly infect hop cultivar also can infect other species like peach, grapevine and citrus. It occurs mostly as mixed infections with other pathogens like with *carlaviruses, Apple mosaic*	[27]

			virus, and *HSVd.* It is als0 found in mixed infection with various fungus species.	
Apscaviroid	Apple dimple fruit viroid (ADFVd)- apple	306	Infects apple cultivars like ‘Royal Gala’, ‘Golden Delicious’, ‘Annurca’ and ‘Red Delicious’, with typical dimpling symptoms (small, a few millimeters in diameter, slightly depressed yellow–green spots scattered on the whole fruit surface and around the calyx). Other symptoms show dappling, crinkling and irregular yellow spots on their fruits.	[28], [29]
	Apple scar skin viroid (ASSVd)^{TS} – apple, dapple apple	329, 331	It causes serious pome fruit diseases such as apple scar skin, dapple apple, pear rusty skin, pear fruit crinkle, and pear dimple fruit.	[30]
	Australian grapevine viroid (AGVd) - grapevine	369	First reported from Australia in 1990. The viroid is reported to be recombinant of many other viroids like citrus exocortis, potato spindle tuber, apple scar skin, and grapevine yellow speckle viroids.	[31]
	Citrus bent leaf viroid (CBLVd) - citrus (Citrus viroid I)	318	Viroid mainly infecting citrus is characterized by bark necrosis and decline. The citrus viroid I (CVd-I) is subgrouped into CVd-Ia and CVd-Ib. CBLVd is the CVd-Ib. The CVd-Ia is known to be derived from the CVd-Ib by the duplication of 5nt in the right terminal region.	[32], [33]
	Citrus dwarfing viroid (CDVd) - IIIa (Citrus viroid IIIa), IIIb (Citrus viroid IIIb)	297,294	Naturally found in citrus and citrus related species. The CDVd has been used to control tree size for the high-density citrus cultivation. The viroid has been reported to be co-infected with other viruses and viroids. CDVd uses the RNA silencing by Citrus tristeza virus (CTV) to accumulate the viroid. Also, new variants of the viroids arising from the point mutations and RNA recombination has also been reported.	[34], [35], [36]
	Citrus viroid V (CVd V) – citrus	294	Naturally infects citrus and citrus related species. Show very small, necrotic, gum-filled lesions on stems. Has shown synergistic interaction with other citrus viroids, so used successfully to identify pathogenic determinant of viroids.	[37], [38]
	Citrus viroid VI - citrus (Citrus viroid-original source, CVdVI-os)	330	First isolated from citrus and persimmon in Japan. Induce symptoms like petiole necrosis and mild leaf bending, and cause less harm when singly infected but in case of the mixed infection with other citrus viruses the loss can be severe.	[39, 40]
	Grapevine yellow speckle viroid (GYSVd-)1, 2 - grapevine	367, 363	Induces the symptoms of small, yellow flecks, scattered over the leaf surface.	[41, 42]

	Pear blister canker viroid – pear (PBCVd)	315	Most pears infected with PBCVd are symptomless, whereas susceptible indicator strains show necrosis on the bark after 3-4 months of infection and dies in 6 months. The viroid RNA is known to be recombinant formed by the segmental exchange of viroid RNAs from same group or other closely related groups.	[43], [44], [45]
	Apple fruit crinkle viroid (AFCVd)*****	371	The viroid RNA majorly infects apples and hop cultivars, also found to be experimentally infectious to tomatoes and cucumbers. Symptoms in apple are crinkling and dappling of mature fruit surface, and the severity of crinkling varies among the commercial apple cultivars. These viroids carry host dependent mutations and the stem loop structure in the left half region is important for the replication.	[46], [47], [48]
Coleviroid	Coleus blumei viroid (CBVd)**TS** 1, 2, 3 - coleus	248, 301,361	CBVd infection primarily affects leaf color of Coleus, the ornamental plant. It shows chlorosis or a purple pigmentation in some varieties of the Coleus plants. The most common way of the transmission of the viroid is by seed transmission, which can be affected by a single nucleotide substitution at the position 25.	[49], [50]

Table S2. The list of linear and circular satRNAs, and a brief description of the relationship of satRNAs with helper virus and the plant host. * - Possible members, satRNA – Satellite RNA, DI-RNA – Defective Interfering RNA, CP-ORF – Coat protein-open reading frame.

	**Linear Sat-RNA**			
**Associated Family or Genus**	**Satellite RNA**	**Genome Size**	**Remarks**	**Ref**
Tombusviridae	Black beet scorch virus (BBSV) satellite RNA	615	The satRNA enhance the pathogenesis and the accumulation of BBSV by alleviating antiviral RNA silencing. The sat-RNA genome is found in multimeric forms, which are used as templates for the replication.	[51-53]
	Cymbidium ringspot virus (CymRSV) satellite RNA	618	The satRNA is parasitic to the helper virus, and replication and accumulation require transacting factors provided by the helper virus and the host. It is known to suppress the accumulation of the CymRSV DI-RNA.	[54-56]
	Panicum mosaic virus (PMV) satellite RNA	826	The satellite PMV elevates the infection by the helper virus by increasing the virus titer. The relationship is determined by two amino acid residues in the CP ORF of PMV.	[57-59]
	Tobacco necrosis virus (TNV) small satellite RNA	629	The satRNA has been patented for the development of the probe for detection of the TNV (PN - JP 1991191784-A/8)	N/A
	Tomato bushy stunt virus (TBSV) satellite RNA	612 (B10), 615 (B1), 822 (Sat-L)	The satRNA B10 significantly reduces the pathogenesis by the helper virus, whereas B1 and Sat-L does not have a significant role in the virus pathogenesis.	[60-62]
Bromoviridae	Cucumber mosaic virus (CMV) satellite RNA	339	The satRNAs have been characterized from more than 25 isolates around the globe and are classified into necrogenic and benign sat-RNAs determined by the necrogenic domain in the 3’ region of the sat-RNA. Depending on the strains of CMV, satellites can alleviate or elevate the symptoms of pathogenesis.	[63, 64]
	Peanut stunt virus (PSV) satellite RNA	393	PSV satRNAs can either exacerbate or attenuate the symptoms caused by the helper virus based on the strains of PSV. Among many strains of the PSV, some	[65, 66]

			strains might not have or support satRNAs at all. Some strains are infact defined by the association of sat-RNAs.	
Genus *Umbravirus*	Carrot mottle mimic virus (CMMV) satellite RNA	748	Tentative evidences have shown that the satRNAS can aggravate the symptoms caused by the virus in *Nicotiana* spp.	[67]
	Groundnut rosette virus (GRV) satellite RNA	901	The satRNA is the actual cause of the rosette symptoms in the Groundnut. A plant host can harbor more than one form of the satRNAs, relative abundance of the variants result in the exhibited symptoms in the host. The satRNA has also been shown to assist the virus in packaging.	[68-70]
	Pea enation mosaic virus (PEMV) satellite RNA	717	The satRNA can exacerbate or alleviate the symptoms caused by the helper virus. Replication and encapsidation of the satRNA is controlled by the RNA2 of the helper virus.	[71]
	Tobacco bushy top virus (TBTV) satellite RNA	779	The satellite-like RNA has been reported but not published. However, most recently, a satRNA-E has been found to cause Ethiopian Tobacco bushy top disease in combination with an Umbravirus-E and Potato leaf roll virus	[72]
	**Circular Sat RNAs**			
Secoviridae	Arabis mosaic virus (AMV) small satellite RNA	300	AMV have a large and small satellite. The smaller satRNA is autocatalytic and known to elevate the symptoms of AMV in the indicator plant, Chenopodium quinoa.	[73, 74]
	Chicory yellow mottle virus (CYMV) satellite RNA	457	Two strains of satRNAs, CYMV-L1 and CYMV-S1 has been reported. L1 satRNAs shows no effects on the pathogenesis by the virus, whereas the S1 satRNA attenuates the symptoms induced by the CYMV in *Nicotiana tabacum.*	[75]
	Tobacco ringspot virus (TRSV) satellite RNA	359	Recognized as molecular parasites to the viral pathogenesis so, have been used to produce disease resistant plants. The satRNA occurs in multimeric forms.	[76, 77]
Luteoviridae	Barley yellow dwarf virus (BYDV) -RPV satellite RNA	322	The satRNA is known to reduce viral RNA accumulation and attenuates symptoms in oats.	[78, 79]

Genus Sobemovirus	Lucerne transient streak virus (LTSV) satellite RNA	324	The replication of the satRNA and its relationship with the helper virus is host dependent. The satRNA is supported by multiple helper viruses like cocksfoor mottle virus and turnip rossette virus. The infectivity by the satRNA is structurally preserved.	[80-82]
	Rice yellow mottle virus (RYMV) satellite	220	It is the smallest satRNA reported. It has the highest sequence similarity with satRNA of LTSV, which are diverse in host range and geographical distribution. The satRNA has not shown effects in the helper virus symptoms modulation or pathogenic relationship with the host plant.	[83, 84]
	Solanum nodiflorum mottle virus (SNMV) satellite RNA	377	The satRNA is known to elevate the symptoms in N. clevelandi and the sat RNA is also supported by distantly related LTSV.	[85, 86]
	Subterranean clover mottle virus (SCMV) satellite RNA (2 types)	332, 388	Can infect plants along with LTSV, a distantly related *Sobemovirus*.	[87]
	Velvet tobacco mottle virus satellite RNA (VTMV)	366	The viroid like satRNA is known to be essential for the infectivity by the helper virus. The satRNA is known to have more affinity for the helper virus replicase leading to favored satRNA replication over the helper virus RNA replication.	[88, 89]
	Cherry small circular viroid-like RNA (cscRNA)*	451	The satRNA has been found in cherry trees infected with the cherry chlorotic rusty spot, a fungal disease. So the cscRNA could be the first mycoviral satRNA.	[90-92]

References

1. Saucedo Carabez, J.R., et al., *The Avocado Sunblotch Viroid: An Invisible Foe of Avocado.* Viruses, 2019. **11**(6): p. 491.
2. Cho, W.K., et al., *A current overview of two viroids that infect chrysanthemums: Chrysanthemum stunt viroid and Chrysanthemum chlorotic mottle viroid.* Viruses, 2013. **5**(4): p. 1099-1113.
3. Jo, Y., et al., *First Report of Peach latent mosaic viroid in Peach Trees in Korea.* Plant Disease, 2016. **100**(1): p. 234.
4. Daros Arnau, J.A., *Eggplant latent viroid: a friendly experimental system in the family Avsunviroidae.* Molecular plant pathology, 2016.
5. Cottilli, P., et al., *Citrus exocortis viroid causes ribosomal stress in tomato plants.* Nucleic acids research, 2019. **47**(16): p. 8649-8661.
6. Imane, B., et al., *Incidence of Citrus exocortis viroid and Hop stund viroid in commercial citrus groves from Morocco.* Moroccan Journal of Agricultural Sciences, 2020. **1**(3).
7. Tangkanchanapas, P., et al., *Whole-Genome Deep Sequencing Reveals Host-Driven in-planta Evolution of Columnea Latent Viroid (CLVd) Quasi-Species Populations.* International journal of molecular sciences, 2020. **21**(9): p. 3262.
8. Bhuvitarkorn, S., S. Klinkong, and K. Reanwarakorn, *Enhancing Columnea latent viroid detection using reverse transcription loop-mediated isothermal amplification (RT-LAMP).* Int. J. Agric. Technol, 2019. **15**: p. 215-228.
9. Spieker, R.L., *The molecular structure of Iresine viroid, a new viroid species from Iresine herbstii ('beefsteak plant').* Journal of General Virology, 1996. **77**(10): p. 2631-2635.
10. Martínez-Soriano, J.P., et al., *Mexican papita viroid: putative ancestor of crop viroids.* Proceedings of the National Academy of Sciences, 1996. **93**(18): p. 9397-9401.
11. Adkar-Purushothama, C.R., et al., *Small RNA Derived from the Virulence Modulating Region of the Potato spindle tuber viroid Silences callose synthase Genes of Tomato Plants.* The Plant Cell, 2015. **27**(8): p. 2178-2194.
12. Mackie, A.E., et al., *Potato spindle tuber viroid: alternative host reservoirs and strain found in a remote subtropical irrigation area.* European Journal of Plant Pathology, 2016. **145**(2): p. 433-446.
13. Katsarou, K., et al., *Insight on genes affecting tuber development in potato upon potato spindle tuber viroid (PSTVd) infection.* PLoS One, 2016. **11**(3): p. e0150711.
14. Adkar-Purushothama, C.R., P.S. Iyer, and J.-P. Perreault, *Potato spindle tuber viroid infection triggers degradation of chloride channel protein CLC-b-like and ribosomal protein S3a-like mRNAs in tomato plants.* Scientific reports, 2017. **7**(1): p. 1-12.
15. Verhoeven, J.T.J., et al., *Tomato apical stunt viroid in the Netherlands: most prevalent pospiviroid in ornamentals and first outbreak in tomatoes.* European journal of plant pathology, 2012. **133**(4): p. 803-810.
16. Matsushita, Y. and S. Tsuda, *Host ranges of Potato spindle tuber viroid, Tomato chlorotic dwarf viroid, Tomato apical stunt viroid, and Columnea latent viroid in horticultural plants.* European journal of plant pathology, 2015. **141**(1): p. 193-197.
17. Batuman, O., et al., *Rasta Disease of Tomato in Ghana is Caused by the Pospiviroids Potato spindle tuber viroid and Tomato apical stunt viroid.* Plant disease, 2019. **103**(7): p. 1525-1535.
18. Matsushita, Y., et al., *First report of a Tomato chlorotic dwarf viroid disease on tomato plants in Japan.* Journal of General Plant Pathology, 2008. **74**(2): p. 182-184.
19. Yanagisawa, H. and Y. Matsushita, *Differences in dynamics of horizontal transmission of Tomato planta macho viroid and Potato spindle tuber viroid after pollination with viroid-infected pollen.* Virology, 2018. **516**: p. 258-264.
20. Maddahian, M., et al., *Biological and molecular characterization of hop stunt viroid variants from pistachio trees in Iran.* Journal of Phytopathology, 2019. **167**(3): p. 163-173.
21. Jo, Y., et al., *Comprehensive analysis of genomic variation of Hop stunt viroid.* European Journal of Plant Pathology, 2017. **148**(1): p. 119-127.
22. Semancik, J. and G. Vidalakis, *The question of Citrus viroid IV as a Cocadviroid.* Archives of virology, 2005. **150**(6): p. 1059-1067.
23. Jakse, J., et al., *Deep-sequencing revealed Citrus bark cracking viroid (CBCV d) as a highly aggressive pathogen on hop.* Plant Pathology, 2015. **64**(4): p. 831-842.

24. Wu, Y., et al., *Characterization of Coconut cadang-cadang viroid variants from oil palm affected by orange spotting disease in Malaysia.* Archives of virology, 2013. **158**(6): p. 1407-1410.
25. Hanold, D. and J. Randles, *Coconut cadang-cadang disease and its viroid agent.* Plant Disease, 1991. **75**(4): p. 330-335.
26. Vadamalai, G., et al., *Coconut Cadang-Cadang Viroid and Coconut Tinangaja Viroid*, in *Viroids and Satellites*. 2017, Elsevier. p. 263-273.
27. Kappagantu, M., et al., *Hop stunt viroid: Effect on host (Humulus lupulus) transcriptome and its interactions with hop powdery mildew (Podospheara macularis).* Molecular Plant-Microbe Interactions, 2017. **30**(10): p. 842-851.
28. Kasai, H., T. Ito, and T. Sano, *Symptoms and molecular characterization of apple dimple fruit viroid isolates from apples in Japan.* Journal of General Plant Pathology, 2017. **83**(4): p. 268-272.
29. Roumi, V., M. Gazel, and K. Caglayan, *First report of apple dimple fruit viroid in apple trees in Iran.* New Dis. Rep, 2017. **35**(3).
30. Hadidi, A., et al., *Apple scar skin viroid*, in *Viroids and Satellites*. 2017, Elsevier. p. 217-228.
31. Rezaian, M.A., *Australian grapevine viroid—evidence for extensive recombination between viroids.* Nucleic Acids Research, 1990. **18**(7): p. 1813-1818.
32. AMIRI, M.M., et al., *Nucleotide Sequence and Structural Features of Hop Stunt Viroid and Citrus Bent Leaf Viroid Variants from Blighted Citrus Plants in Kohgiluyeh–Boyerahmad Province of Iran.* 2014.
33. Hataya, T., et al., *Citrus viroid Ia is a derivative of citrus bent leaf viroid (CVd-Ib) by partial sequence duplications in the right terminal region.* Archives of virology, 1998. **143**(5): p. 971-980.
34. Murcia, N., et al., *Molecular and biological characterization of natural variants of Citrus dwarfing viroid.* Archives of virology, 2009. **154**(8): p. 1329-1334.
35. Serra, P., et al., *Virus-Viroid Interactions: Citrus Tristeza Virus Enhances the Accumulation of Citrus Dwarfing Viroid in Mexican Lime via Virus-Encoded Silencing Suppressors.* Journal of Virology, 2014. **88**(2): p. 1394-1397.
36. Owens, R., et al., *Both point mutation and RNA recombination contribute to the sequence diversity of citrus viroid III.* Virus Genes, 2000. **20**(3): p. 243-252.
37. Serra, P., et al., *Citrus viroid V: molecular characterization and synergistic interactions with other members of the genus Apscaviroid.* Virology, 2008. **370**(1): p. 102-112.
38. Serra, P., et al., *An artificial chimeric derivative of Citrus viroid V involves the terminal left domain in pathogenicity.* Molecular plant pathology, 2009. **10**(4): p. 515-522.
39. Ito, T., et al., *Characterization of a new citrus viroid species tentatively termed Citrus viroid OS.* Arch Virol, 2001. **146**(5): p. 975-82.
40. Ito, T., et al., *Multiple Citrus Viroids in Citrus from Japan and Their Ability to Produce Exocortis-Like Symptoms in Citron.* Phytopathology®, 2002. **92**(5): p. 542-547.
41. Koltunow, A.M. and M.A. Rezaian, *Grapevine yellow speckle viroid: structural features of a new viroid group.* Nucleic Acids Research, 1988. **16**(3): p. 849-864.
42. Koltunow, A.M., et al., *Two Related Viroids Cause Grapevine Yellow Speckle Disease Independently.* Journal of General Virology, 1989. **70**(12): p. 3411-3419.
43. Joyce, P., et al., *Characterisation of Pear blister canker viroid isolates from Australian pome fruit orchards.* Australasian Plant Pathology, 2006. **35**(4): p. 465-471.
44. Ambrós, S., et al., *Pear blister canker viroid: sequence variability and causal role in pear blister canker disease.* Journal of General Virology, 1995. **76**(10): p. 2625-2629.
45. Hernández, C., et al., *Pear blister canker viroid is a member of the apple scar skin subgroup (apscaviroids) and also has sequence homology with viroids from other subgroups.* Journal of General Virology, 1992. **73**(10): p. 2503-2507.
46. ITO, T., et al., *Detection of a viroid associated with apple fruit crinkle disease.* Japanese Journal of Phytopathology, 1993. **59**(5): p. 520-527.
47. Sano, T., et al., *Vegetative propagation and its possible role as a genetic bottleneck in the shaping of the apple fruit crinkle viroid populations in apple and hop plants.* Virus Genes, 2008. **37**(3): p. 298-303.

48. Suzuki, T., et al., *Characterization of host-dependent mutations of apple fruit crinkle viroid replicating in newly identified experimental hosts suggests maintenance of stem–loop structures in the left-hand half of the molecule is important for replication.* Journal of General Virology, 2017. **98**(3): p. 506-516.
49. Chung, B.N. and G.S. Choi, *Incidence of Coleus blumei viroid 1 in seeds of commercial Coleus in Korea.* Plant Pathol. J, 2008. **24**(3): p. 305-308.
50. Tsushima, T. and T. Sano, *A point-mutation of Coleus blumei viroid 1 switches the potential to transmit through seed.* Journal of General Virology, 2018. **99**(3): p. 393-401.
51. Guo, L.-H., et al., *Analysis of Nucleotide Sequences and Multimeric Forms of a Novel Satellite RNA Associated with Beet Black Scorch Virus.* Journal of Virology, 2005. **79**(6): p. 3664–3674
52. Xu, J., et al., *Improved Pathogenicity of a Beet Black Scorch Virus Variant by Low Temperature and Co-infection with Its Satellite RNA.* Frontiers in Microbiology, 2016. **7**: p. 1771
53. Xu, J., et al., *Two distinct sites are essential for virulent infection and support of variant satellite RNA replication in spontaneous beet black scorch virus variants.* Journal of General Virology, 2012. **93**(12): p. 2718-2728.
54. Pantaleo, V. and J. Burgyán, *Cymbidium Ringspot Virus Harnesses RNA Silencing To Control the Accumulation of Virus Parasite Satellite RNA.* Journal of Virology, 2008. **82**(23): p. 11851–11858
55. Rubino, L., et al., *Sequence analysis of cymbidium ringspot virus satellite and defective interfering RNAs.* The Journal of general virology, 1990. **71 (Pt 8)**: p. 1655-60.
56. Rubino, L., J.C. Carrington, and M. Russo, *Biologically active cymbidium ringspot virus satellite RNA in transgenic plants suppresses accumulation of DI RNA.* Virology, 1992. **188**(2): p. 429–437.
57. Chowda-Reddy, R.V., et al., *A Two-Amino Acid Difference in the Coat Protein of Satellite panicum mosaic virus Isolates Is Responsible for Differential Synergistic Interactions with Panicum mosaic virus.* Molecular Plant-Microbe Interactions®, 2019. **32**(4): p. 479-490.
58. Desvoyes, B. and K.-B.G. Scholthof, *RNA: protein interactions associated with satellites of panicum mosaic virus.* FEBS Letters, 2000. **485**(1): p. 25 - 28
59. Masuta, C., et al., *Analysis of the genome of satellite panicum mosaic virus.* Virology, 1987. **159**(2): p. 329 - 338
60. Célix, A., J. Burgyán, and E. Rodríguez-Cerezo, *Interactions between Tombusviruses and Satellite RNAs of Tomato Bushy Stunt Virus: A Defect in sat RNA B1 Replication Maps to ORF1 of a Helper Virus.* Virology, 1999. **262**(1): p. 129 - 138
61. Celix, A., E. Rodriguez-Cerezo, and F. Garcia-Arenal, *New Satellite RNAs, but No DI RNAs, Are Found in Natural Populations of Tomato Bushy Stunt Tombusvirus.* Virology, 1997. **239**(2): p. 277 - 284.
62. Rubino, L. and M. Russo, *Properties of a novel satellite RNA associated with tomato bushy stunt virus infections.* Journal of General Virology, 2010. **91**(9): p. 2393-2401
63. Devic, M., M. Jaegle, and D. Baulcombe, *Cucumber mosaic virus satellite RNA (strain Y): analysis of sequences which affect systemic necrosis on tomato.* Journal of General Virology, 1990. **71**(7): p. 1443-1449
64. Grieco, F., et al., *Nucleotide sequence of a cucumber mosaic virus satellite RNA associated with a tomato top stunting.* Nucleic acids research, 1992. **20**(24): p. 6733
65. Milit√£o, V., et al., *Differential interactions among isolates of peanut stunt cucumovirus and its satellite RNA.* Journal of General Virology, 1998. **79**(1): p. 177-184
66. Obrępalska-Stęplowska, A., et al., *The Defense Response of Nicotiana benthamiana to Peanut Stunt Virus Infection in the Presence of Symptom Exacerbating Satellite RNA.* Viruses, 2018. **10**(9).
67. Menzel, W., E. Maiss, and H.J. Vetten, *Nucleotide sequence of a satellite RNA associated with carrot motley dwarf in parsley and carrot.* Virus genes, 2009. **38**(1): p. 187-8.
68. Blok, V.C., et al., *Sequences of 10 Variants of the Satellite-like RNA-3 of Groundnut Rosette Virus.* Virology, 1994. **202**(1): p. 25 - 32
69. Murant, A.F. and T.l.I.K. Kumar, *Different variants of the satellite RNA of groundnut rosette virus are responsible for the chlorotic and green forms of groundnut rosette disease*.* Annals of Applied Biology, 1990. **117**(1): p. 85-92
70. Robinson, D.J., et al., *Satellite RNA Is Essential for Encapsidation of Groundnut Rosette Umbravirus RNA by Groundnut Rosette Assistor Luteovirus Coat Protein.* Virology, 1999. **254**(1): p. 105 - 114

71. Demler, S.A., et al., *Replication of the satellite RNA of pea enation mosaic virus is controlled by RNA 2-encoded functions.* Journal of General Virology, 1994. **75**(6): p. 1399-1406.
72. Abraham, A.D., et al., *A novel combination of a new umbravirus, a new satellite RNA and potato leafroll virus causes tobacco bushy top disease in Ethiopia.* Archives of virology, 2014. **159**(12): p. 3395-3399.
73. Kaper, J.M., M.E. Tousignant, and G. Steger, *Nucleotide sequence predicts circularity and self-cleavage of 300-ribonucleotide satellite of arabis mosaic virus.* Biochemical and Biophysical Research Communications, 1988. **154**(1): p. 318 - 325
74. Etscheid, M., M.E. Tousignant, and J.M. Kaper, *Small satellite of arabis mosaic virus: autolytic processing of in vitro transcripts of (+) and (-) polarity and infectivity of (+) strand transcripts.* Journal of General Virology, 1995. **76**(2): p. 271-282.
75. Rubino, L., et al., *Nucleotide sequence and structural analysis of two satellite RNAs associated with chicory yellow mottle virus.* The Journal of general virology, 1990. **71 (Pt 9)**: p. 1897-903.
76. Buzayan, J.M., et al., *Nucleotide sequence of satellite tobacco ringspot virus RNA and its relationship to multimeric forms.* Virology, 1986. **151**(2): p. 186-99.
77. Robaglia, C., et al., *Evolution and replication of tobacco ringspot virus satellite RNA mutants.* The EMBO Journal, 1993. **12**(7): p. 2969-2976
78. Miller, W.A., et al., *A satellite RNA of barley yellow dwarf virus contains a novel hammerhead structure in the self-cleavage domain.* Virology, 1991. **183**(2): p. 711-20.
79. Rasochová, L., et al., *The Satellite RNA of Barley Yellow Dwarf Virus-RPV Is Supported by Beet Western Yellows Virus in Dicotyledonous Protoplasts and Plants.* Virology, 1997. **231**(2): p. 182 - 191
80. Gellatly, D., et al., *Structural and sequence integrity are essential for the replication of the viroid-like satellite RNA of lucerne transient streak virus.* Journal of General Virology, 2011. **92**(6): p. 1475-1481.
81. Keese, P., G. Bruening, and R.H. Symons, *Comparative sequence and structure of circular RNAs from two isolates of lucerne transient streak virus.* FEBS Letters, 1983. **159**(1): p. 185 - 190
82. Sehgal, O.P., et al., *Replication and encapsidation of the viroid-like satellite RNA of lucerne transient streak virus are supported in divergent hosts by cocksfoot mottle virus and turnip rosette virus.* Journal of General Virology, 1993. **74**(4): p. 785-788.
83. Collins, R.F., et al., *Self-cleaving circular RNA associated with rice yellow mottle virus is the smallest viroid-like RNA.* Virology, 1998. **241**(2): p. 269-75.
84. Pinel, A., et al., *Molecular epidemiology of the RNA satellite of Rice yellow mottle virus in Africa.* Archives of virology, 2003. **148**(9): p. 1721–1733.
85. Jones, A.T. and M.A. Mayo, *Interaction of Lucerne Transient Streak Virus and the Viroid-like RNA-2 of Solanum nodiflorum Mottle Virus.* Journal of General Virology, 1983. **64**(8): p. 1771-1774.
86. Haseloff, J. and R.H. Symons, *Comparative sequence and structure of viroid-like RNAs of two plant viruses.* Nucleic Acids Res, 1982. **10**(12): p. 3681-91.
87. Dall, D.J., et al., *Isolation of a Subterranean Clover Mottle Virus-like Satellite RNA from Lucerne Infected with Lucerne Transient Streak Virus.* Journal of General Virology, 1990. **71**(8): p. 1873-1875
88. Francki, R.I.B., C.J. Grivell, and K.S. Gibb, *Isolation of velvet tobacco mottle virus capable of replication with and without a viroid-like RNA.* Virology, 1986. **148**(2): p. 381 - 384
89. Hanada, K. and R.I.B. Francki, *Kinetics of velvet tobacco mottle virus satellite RNA synthesis and encapsidation.* Virology, 1989. **170**(1): p. 48 - 54
90. Di Serio, F., et al., *A 451-nucleotide circular RNA from cherry with hammerhead ribozymes in its strands of both polarities.* Journal of virology, 1997. **71**(9): p. 6603-10.
91. Di Serio, F., et al., *Close structural relationship between two hammerhead viroid-like RNAs associated with cherry chlorotic rusty spot disease.* Archives of virology, 2006. **151**(8): p. 1539–1549.
92. Minoia, S., et al., *Viroid-like RNAs from cherry trees affected by leaf scorch disease: further data supporting their association with mycoviral double-stranded RNAs.* Archives of virology, 2014. **159**(3): p. 589-93.
